# Hypergonadotropic Hypogonadism Due to Transaldolase Deficiency: Two Cases and Literature Review

**DOI:** 10.1210/jcemcr/luae028

**Published:** 2024-03-04

**Authors:** Akram Takaleh, Nasser Abunamous, Aisha AlShamsi, Noura Alhassani, Raya Almazrouei

**Affiliations:** Department of Internal Medicine, Tawam Hospital, Al Ain, United Arab Emirates; Department of Internal Medicine, Tawam Hospital, Al Ain, United Arab Emirates; Division of Genetics and Metabolic, Department of Paediatrics, Tawam Hospital, Al Ain, United Arab Emirates; Division of Endocrine and Diabetes, Department of Pediatrics, Tawam Hospital; Department of Paediatrics, College of Medicine and Health Science, United Arab Emirates University, Al Ain, United Arab Emirates; Division of Endocrinology, Department of Internal Medicine, Tawam Hospital, Al Ain, United Arab Emirates; Department of Internal Medicine, College of Medicine and Health Sciences, United Arab Emirates University, Al Ain, United Arab Emirates

**Keywords:** transaldolase deficiency, hypergonadotropic hypogonadism, POI, *TALDO1*, delayed puberty

## Abstract

Transaldolase deficiency is a rare autosomal recessive inborn error of carbohydrate metabolism caused by pathogenic/likely pathogenic biallelic mutations in the *TALDO1* gene. This disorder is characterized by multisystem involvement with variable phenotypes, including intrauterine growth restriction; dysmorphic features; abnormal skin; hepatosplenomegaly; cytopenia; and cardiac, renal, and endocrine abnormalities. Herein, we present two Emirati patients with hypergonadotropic hypogonadism due to transaldolase deficiency and variable phenotypes of systemic involvement.

## Introduction

Transaldolase deficiency (OMIM 606003) is an inborn error in the nonoxidative phase of the pentose phosphate pathway (PPP). The PPP consists of oxidative and nonoxidative components, which generate nicotinamide adenine dinucleotide phosphate (NADPH), used for biosynthetic reactions and neutralization of reactive oxygen intermediates, and ribose, used for the synthesis of ribonucleic acid, deoxyribonucleic acid, and adenosine triphosphate. The PPP presents in several tissues, such as the lung, liver, mammary glands, ovaries, pituitary gland, testis, thyroid gland, brain, adrenal cortex, and skin (1). Transaldolase is the second enzyme in the nonoxidative component. In the absence of this enzyme, polyols accumulate in body fluids, mostly in the urine, and failure to recycle ribose 5-phosphate occurs, which leads to NADPH and glutathione deficiency (2). Transaldolase deficiency was first described in 2001 in a patient with prominent liver involvement in infancy (3). It is an autosomal recessive disorder caused by biallelic pathogenic mutations in the *TALDO1* gene. It has a wide clinical phenotype, including hydrops fetalis; intrauterine growth restriction; short stature with failure to thrive; dysmorphic features; dry, scaly skin; hepatosplenomegaly; cytopenia; and renal, respiratory, and cardiac involvement. Patients may have early-onset disease with severe symptoms during the neonatal period, which may lead to death or a relatively mild late-onset presentation (4‐7). In addition to short stature, endocrine evaluation in patients with transaldolase deficiency may reveal gonadal dysfunction with hypergonadotropic hypogonadism. This has been recognized recently with few case reports in the literature, mainly of Turkish and Arabic origins (8, 9). Our two reported patients here are added to the scarce existing knowledge about this rare cause of hypergonadotropic hypogonadism. The first patient mentioned in this case report was one of four Emirati patients previously reported with *TALDO1* gene mutation (10).

## Case Presentation

### Patient 1

A 32-year-old woman was seen in the endocrine clinic at the age of 25 years for secondary amenorrhea evaluation. The patient had her menarche at the age of 17 years with light menses of 1 to 2 times per year, which then ceased completely at the age of 20 years. The patient denied hot flushes and mucosal dryness. Her family history was significant for her parents being first-degree cousins, and the patient had two siblings who died after birth for unknown causes (one immediately after birth and another one at the age of 6 months). Her medical history was significant for failure to thrive with poor weight gain, long-standing hepatomegaly discovered after birth (progressed over time to liver cirrhosis), and bicytopenia (thrombocytopenia and leukopenia). During childhood, thorough investigations, including muscle and scalp hair biopsies, were inconclusive. Bone marrow aspiration and liver biopsy were declined by the family. Since then, the patient has been lost to follow-up for many years, and at the age of 25 years, the patient was evaluated by the genetic team after her nephew was diagnosed with transaldolase deficiency. The patient was also referred for endocrine service to evaluate secondary amenorrhea.

### Patient 2

A 17-year-old boy with short stature was seen in the endocrine clinic at the age of 9 years. His medical history was significant for premature birth with a birth weight of 1.7 kg. The patient was admitted to the neonatal intensive care unit for 1 month. At the age of 3 weeks, the patient underwent bilateral orchiopexy, hernia repair, and circumcision. Furthermore, the patient had recurrent chest infections in the first three years of life, failure to thrive, and short stature. Moreover, mild hepatomegaly and leukopenia were observed with no clear underlying cause. His family history was significant because his parents were first-degree cousins, and the patient had a sister with recurrent chest infections who was diagnosed with possible cystic fibrosis, although her cystic fibrosis transmembrane conductance regulator (*CFTR)* gene sequencing was negative. Furthermore, the patient's sister had dilated cardiomyopathy and died at the age of 13 years from an upper gastrointestinal bleed.

## Diagnostic Assessment

### Patient 1

At the age of 25 years, the patient had a low body weight of 38.8 kg, a height of 157 cm, and a body mass index of 15.74 kg/m^2^. She had an elongated, thin face with horizontal nystagmus. Skin examination revealed ichthyosis with diffuse dry, scaly skin. Abdominal examination revealed hepatomegaly and a normal-sized spleen. On assessment of puberty, the patient was at Tanner stage III for breast, whereas the Tanner stage for pubic hair development was not documented. The results of the investigations are shown in Table 1. Regarding amenorrhea, the hormonal panel was suggestive of hypergonadotropic hypogonadism. Furthermore, laboratory investigation showed microalbuminuria (60 mg/L) and stage II chronic kidney disease with an estimated glomerular filtration rate of 66 mL/min/1.73 m^2^. Karyotype showed normal female 46XX chromosomes, and 21-hydroxylase antibody level was not determined. Transabdominal pelvic ultrasonography (US) and echocardiography findings are depicted in Table 1. Electrocardiogram showed incomplete right bundle branch block. Further investigations included urine polyols (Table 2), which revealed significantly elevated levels of sedoheptulose, ribitol, and arabitol, suggesting transaldolase deficiency. Molecular analysis revealed a *TALDO1* (exon 5)–pathogenic homozygous mutation (*c.574C > T p.Arg192Cys*), confirming the diagnosis of transaldolase deficiency.

**Table 1.** Laboratory profile and ultrasound abdomen scan findings in both patients

Lab	Patient 1	Patient 2	Reference range
White blood cell count	2900/µL (2.9 ×10^9/L)	3500/µL (3.5 ×10^9/L)	4500-11000/µL (4.5-11.0 ×10^9/L)
Neutrophil count	1590/µL (1.59 ×10^9/L)	1800/µL (1.8 ×10^9/L)	1800- 7700/µL (1.80-7.70 ×10^9/L)
Hemoglobin	11.7 g/dL (117 g/L)	10.9 g/dL (109 g/L)	11.7-15.5 g/dL (117-155 g/L)
Platelets	45 × 10^3^/µL (45 ×10^9/L)	292 × 10^3^/µL (292 ×10^9/L)	140-450 × 10^3^/µL (140-450 ×10^9/L)
AST	33 IU/L (0.55 µkat/L)	30 IU/L (0.5 µkat/L)	15-41 IU/L (0.25-0.68 µkat/L)
ALT	23 IU/L (0.38 µkat/L)	22 IU/L (0.37 µkat/L)	14-54 IU/L (0.23-0.9 µkat/L)
GGT	33 IU/L (0.55 µkat/L)	14 IU/L (0.23 µkat/L)	9-35 IU/L (0.15-0.58 µkat/L)
ALP	90 IU/L (1.5 µkat/L)	235 IU/L (3.92 µkat/L)	38-126 IU/L (0.63-2.1 µkat/L)
Albumin	4.6 g/dL (46 g/L)	4.4 g/dL (44 g/L)	3.5-4.8 g/dL (35-48 g/L)
Total bilirubin	0.99 mg/dL (17 μmol/L)	0.69 mg/dL (11.8 μmol/L)	0.29-1.23 mg/dL (5-21 μmol/L)
Direct bilirubin	0.11 mg/dL (1.8 μmol/L)	0.13 mg/dL (2.3 μmol/L)	0.01-0.50 mg/dL (1.7-8.6 μmol/L)
INR	1.08	1.21	< 1.1
Prothrombin time	11.6 seceonds	13 seceonds	11-13.5 seceonds
LH	17.26 mIU/mL (17.26 IU/L)	58.6 mIU/mL (58.6 IU/L)	Adult Female range 5-25 mIU/mL (5-25 IU/L) Male pubertal range 1.24-7.8 mIU/mL (1.24-7.8 IU/L)
FSH	23.21 mIU/mL (23.21 IU/L)	152 mIU/mL (152 IU/L)	Adult Female range 4.5-21.5 mIU/mL (4.5-21.5 IU/L) Male pubertal range 1.5-12.4 mIU/mL (1.5-12.4 IU/L)
Estradiol	19.86 pg/mL (<73 pmol/L)	—	Adult Female range 29.92-399.29 pg/mL (110-1468 pmol/L)
Total testosterone	12.1 ng/dL (0.42 nmol/L)	9.2 ng/dL (0.32 nmol/L)	Male pubertal range 288-1008 ng/dL (10-35 nmol/L)
Ultrasound abdomen	Enlarged liver, abnormal position, granular pattern, with no focal lesions. The portal vein shows abnormal pattern with suspicion of right portal vein agenesis and secondary right liver lobe agenesis. The spleen is normal in echogenicity and size.	Enlarged liver, no focal lesions, normal portal vein, and spleen is normal in size.	—
Ultrasound pelvis/scrotum	Anteverted uterus measuring 6.32 × 2.2 × 3.19 cm with a thin endometrium and left ovary measuring 1.6 × 1.25 × 1.31 cm; the right ovary was not seen.	Small bilateral testes (prepubertal) with sizes of 1 × 0.8 × 0.5 cm for the right testis and 1.8 × 1.1 × 0.46 cm for the left testis.	—
Echocardiography	Bicuspid aortic valve without other chamber abnormalities.	Normal cardiac structures, left ventricular ejection fraction of 67%, normal systolic and diastolic function, and trace aortic regurgitation.	—

Abbreviations: ALP, alkaline phosphatase; ALT, alanine aminotransferase; AST, aspartate aminotransferase; GGT, γ-glutamyltransferase; INR, international normalized ratio.

Values in parenthesis are International System of Units (SI).

**Table 2.** Urine polyol determination in patient 1

Polyol	Results (mmol/mol creatinine)	Reference range (mmol/mol creatinine)
Arabitol	140.9 (H)	(32-88)
Erythritol	146.5	(55-192)
Ribitol	42.2 (H)	(8-24)
Xylitol	1.4 (L)	(5-49)
Galactitol	1.7 (L)	(3-81)
Sedoheptulose	553.4 (H)	<1
Sedoheptitol	0	<1

Abbreviations: H, high; L, low.

### Patient 2

At the age of 9 years, the patient had a low body weight of 16.9 ± 4.77 kg, height of 117.5 ± 2.84 cm, and body mass index of 12.24 ± 3.89 kg/m^2^ with a midparental height of 175 cm. Skin examination revealed dry, scaly skin, cutaneous telangiectasia, and hyperpigmentation. Cardiac examination revealed a soft ejection systolic murmur at the left lower sternal border. The patient was prepubertal with normal genitalia for age and sex and Tanner stage I with bilateral palpable but insignificant testes of 1 mL in volume. Because the patient's growth velocity (GV) was poor, a GH stimulation test was performed using oral clonidine at a dose of 0.15 mg/m^2^ and intramuscular injection of glucagon at a dose of 0.03 mg/kg after an overnight fast according to our center protocol. The peak GH level was 23.44 micog/L, indicating no GH deficiency. The results of the other investigations are shown in Table 1. Apart from leukopenia, the hormonal panel (performed at the age of 14 years for delayed puberty) indicated hypergonadotropic hypogonadism. Karyotype revealed a normal male karyotype, 46XY. Scrotal US revealed small bilateral testes (prepubertal). Abdominal US and echocardiography findings are shown in Table 1. With multisystem involvement, the patient was followed up in different subspecialty clinics during childhood, including gastroenterology and pulmonary, without a clear cause identified. At the age of 10 years, the patient was referred to the genetic team after discussion of being from the same tribe of previously identified transaldolase deficiency cases. Molecular analysis revealed a homozygous mutation (*c.574C > T p.Arg192Cys*) in *TALDO1*, confirming the diagnosis of transaldolase deficiency.

## Treatment

### Patient 1

Hormonal replacement therapy in the form of an oral combination of estradiol valerate 2 mg and norgestrel 0.5 mg was initiated; however, the patient was not compliant and was lost to follow-up for years.

### Patient 2

Initially, the patient was supported with nutritional supplements (formula of Ensure Plus, 2-3 bottles per day) while being kept under GV observation. However, at the age of 11 years, as his height was still poor (−2.7 SDS), while his weight was −4.6 SDS, poor GV 2.6 cm/year, and delayed bone age at −2.2 SDS with predicted final adult height of 160 cm, which was below his midparental height, it was agreed to start GH treatment as a case of idiopathic short stature.

## Outcome and Follow-up

### Patient 1

At the age of 29 years, the patient developed recurrent spontaneous left-sided pneumothorax for which she underwent video-assisted thoracoscopic surgery, bullectomy, and mechanical pleurodesis. In addition to pneumothorax, computed tomography of the chest revealed multiple bilateral peripheral subpleural emphysematous changes and emphysematous bullae. The patient was followed up regularly in the pulmonary and thoracic clinics with stable findings. At the age of 31 years, the patient was assessed again in the endocrine clinic, and dual-energy X-ray absorptiometry revealed very low bone density below the expected age range (lumbar Z-score, −3.5; left femur Z-score, −2.9; right femur Z-score, −2.7). Hormone replacement therapy [estradiol patch (50 mcg/day) twice weekly and micronized progesterone of 200 mg for 14 days per month] was reinitiated.

### Patient 2

After the first year of GH treatment, the patient's height improved to −2.2 SDS with an increase in GV to approximately 8 cm/year. Currently, the patient is 17 years of age and is still on GH injections with a height of 167 cm at −1.1 SDS, although his weight remains poor at −3 SDS. The latest bone age is still delayed at −2.2 SDS. At the age of 14 years, puberty induction was initiated for the diagnosis of hypergonadotropic hypogonadism with monthly testosterone ester injections (currently in Tanner stage III), and the patient is still being followed up in the endocrine clinic.

## Discussion

In this report, we described two patients with multisystem involvement since the neonatal–childhood period with unknown etiology who were additionally diagnosed with hypergonadotropic hypogonadism in adolescence. Several years later, both patients were found to have a homozygous mutation in the *TALDO1* gene, causing transaldolase deficiency.

In a recent multicenter survey that involved 34 patients from 21 families (22 with early-onset disease and 12 with late-onset disease), abnormal external genitalia were described in 11 (32%) patients (36% in those with early-onset disease and 25% in those with late-onset disease). The described abnormalities included 3 males with small phallus, 3 females with clitoromegaly, and 6 males with cryptorchidism. Furthermore, hypergonadotropic hypogonadism was described in 7 (18%) patients, and all patients had early-onset disease. However, it is worth mentioning that most patients in this survey were young, with a median age of 6 years at the last follow-up or death. Only 1 patient in that survey reached adulthood (7). Hypergonadotrophic hypogonadism with late-onset disease was recently described in one male patient (11). Our first patient had delayed spontaneous puberty that could be described as partial or with early arrest because she remained amenorrheic with 1 to 2 scanty periods per year and clinically at the early Tanner stage. However, the second patient did not begin puberty on normal timing with no testicular enlargement. These differences agree with those of a previous survey study that showed variability in phenotype and no genotype–phenotype correlation (7).

The mechanism underlying gonadal dysfunction is not fully understood. Several hypotheses have been proposed. Transaldolase activity is required for nucleic acid production, NADPH synthesis, and the reduction of cellular reactive oxygen species (ROS). One hypothesis is that the NADPH/NADP ratio contributes to most hormone biosynthetic pathways, and the decreased NADPH in transaldolase deficiency could be a cause of abnormal steroid hormone production (7). However, increased levels of ROS and toxic accumulation of C5 polyols (xylitol) and seven-carbon sugars may cause gonadal cell apoptosis and damage (2). In one case, ROS accumulation was measured using 2′,7′-dichlorodihydrofluorescein diacetate in fibroblasts from a patient with transaldolase deficiency and healthy controls. The affected patient's fibroblasts showed significantly increased accumulation of ROS (207%, *P* = .007), compared with controls, indicating an increased susceptibility to oxidative stress (8).

Short stature was reported in a few patients, whereas most patients were not evaluated for this parameter. It is speculated that short stature in some patients is correlated with disease severity and comorbidities because chronic liver, cardiac, and renal illnesses may contribute to poor growth. In contrast, poor nutrition may decrease insulin-like growth factor 1 levels (7). Our first patient had no issue with stature; therefore, GH levels were not evaluated, In the second patient, however, the short stature was attributed to prematurity, intrauterine growth restriction, malnutrition, and systemic illness. The formal GH stimulation test for this patient showed a normal response; however, GH treatment was initiated because of poor height velocity.

Currently, no specific treatment for transaldolase deficiency exists. Replacing the affected organ, such as the liver, with a transplant would be an option in patients with end-stage liver disease. Theoretically, there could be a rule for antioxidants, although this should be investigated. Hypergonadotropic hypogonadism should be managed with sex hormone replacement. Whether early diagnosis provides the opportunity for tissue preservation for future fertility should be considered.

## Learning Points

- Transaldolase deficiency may be considered in the differential diagnosis of patients with delayed puberty due to hypergonadotropic hypogonadism, mainly in the presence of an unknown cause of hepatomegaly or cirrhosis along with multisystem involvement.
- Because no specific treatment or enzyme replacement exists for the disease, these patients require long-term follow-up of gonadal function along with follow-up of other organ-specific manifestations.
- Although puberty may start spontaneously, patients may still be at risk of developing hypergonadotropic hypogonadism later in life.

## Data Availability

Data sharing is not applicable to this article as no datasets were generated or analyzed during the current study.
